# Supplementary material for: Route to chaos and resonant triads interaction in a truncated rotating nonlinear shallow–water model
Source: PLoS One. 2024 Aug 9;19(8):e0305534. doi: 10.1371/journal.pone.0305534 (PMC11315320; doi:10.1371/journal.pone.0305534)
Supplement: S1 File — (ZIP) [file pone.0305534.s001.zip › SupportingInformation.pdf]

Supporting Information

Francesco Carbone[1] and Denys Dutykh[2,3]

[1]National Research Council - Institute of Atmospheric Pollution Research, C/o University of Calabria, 87036 Rende, Italy
[2]Mathematics Department, Khalifa University of Science and Technology, PO Box 127788, Abu Dhabi, United Arab Emirates
[3]Causal Dynamics Pty Ltd, Perth, Australia

July 12, 2024

S1: Supporting information

S1.1: Truncated RSW $\mathcal{T}_5$ system

The system of equations describing the temporal evolution of the $\mathcal{T}_5(u, v, \eta)$ system, with $u_k(t), v_k(t), \eta_k(t) \in \mathbb{C}$ ($k \in [1, 2, \ldots, 5]$):

$$\frac{\mathrm{d}u_1}{\mathrm{d}t} = -\mathrm{i}\frac{k_0}{2}\Big[-v_3 u_2^\star + 2u_3 v_2^\star\Big] + \frac{v_1}{\mathrm{Ro}} - \frac{k_0^2}{\mathrm{Re}} u_1 \tag{1}$$

$$\begin{aligned}\frac{\mathrm{d}u_2}{\mathrm{d}t} &= -\mathrm{i}k_0\eta_2 - \mathrm{i}\frac{k_0}{2}\Big[u_3 u_1^\star - v_3 u_1^\star + 2u_3 v_1^\star + u_5 u_4^\star + v_5 u_4^\star\Big] + \\ &+ \frac{v_2}{\mathrm{Ro}} - 2\frac{k_0^2}{\mathrm{Re}} u_2\end{aligned} \tag{2}$$

$$\frac{\mathrm{d}u_3}{\mathrm{d}t} = -\mathrm{i}k_0\eta_3 - \mathrm{i}\frac{k_0}{2}\Big[u_2 u_1 + v_2 u_1 + u_2 v_1\Big] + \frac{v_3}{\mathrm{Ro}} - 5\frac{k_0^2}{\mathrm{Re}} u_3 \tag{3}$$

$$\frac{\mathrm{d}u_4}{\mathrm{d}t} = -2\mathrm{i}k_0\eta_4 - \mathrm{i}\frac{k_0}{2}\Big[u_5 u_2^\star - v_5 u_2^\star\Big] + \frac{v_4}{\mathrm{Ro}} - 5\frac{k_0^2}{\mathrm{Re}} u_4 + F_u \tag{4}$$

$$\frac{\mathrm{d}u_5}{\mathrm{d}t} = -3\mathrm{i}k_0\eta_5 - \mathrm{i}\frac{k_0}{2}\Big[3u_4 u_2 + v_4 u_2 - u_4 v_2\Big] + \frac{v_5}{\mathrm{Ro}} - 9\frac{k_0^2}{\mathrm{Re}} u_5 \tag{5}$$

$$\frac{\mathrm{d}v_1}{\mathrm{d}t} = -\mathrm{i}k_0\eta_1 - \mathrm{i}\frac{k_0}{2}\Big[v_3v_2^\star - u_3v_2^\star + v_3u_2^\star\Big] - \frac{u_1}{\mathrm{Ro}} - \frac{k_0^2}{\mathrm{Re}}v_1 \quad (6)$$

$$\frac{\mathrm{d}v_2}{\mathrm{d}t} = -\mathrm{i}k_0\eta_2 - \mathrm{i}\frac{k_0}{2}\Big[v_3v_1^\star + v_3u_1^\star + v_5v_4^\star - 2u_5v_4^\star + 3v_5u_4^\star\Big] - $$

$$- \frac{u_2}{\mathrm{Ro}} - 2\frac{k_0^2}{\mathrm{Re}}v_2 \quad (7)$$

$$\frac{\mathrm{d}v_3}{\mathrm{d}t} = -2\mathrm{i}k_0\eta_3 - \mathrm{i}\frac{k_0}{2}\Big[2v_2v_1 + v_2u_1\Big] - \frac{u_3}{\mathrm{Ro}} - 5\frac{k_0^2}{\mathrm{Re}}v_3 \quad (8)$$

$$\frac{\mathrm{d}v_4}{\mathrm{d}t} = \mathrm{i}k_0\eta_4 - \mathrm{i}\frac{k_0}{2}\Big[-v_5v_2^\star - u_5v_2^\star + 3v_5u_2^\star\Big] - \frac{u_4}{\mathrm{Ro}} - 5\frac{k_0^2}{\mathrm{Re}}v_4 \quad (9)$$

$$\frac{\mathrm{d}v_5}{\mathrm{d}t} = -\mathrm{i}\frac{k_0}{2}\Big[u_4v_2 + 2v_4u_2\Big] - \frac{u_5}{\mathrm{Ro}} - 9\frac{k_0^2}{\mathrm{Re}}v_5 \quad (10)$$

$$\frac{\mathrm{d}\eta_1}{\mathrm{d}t} = -\mathrm{i}k_0v_1 - \mathrm{i}\frac{k_0}{2}\Big[\eta_3v_2^\star + v_3\eta_2^\star\Big] \quad (11)$$

$$\frac{\mathrm{d}\eta_2}{\mathrm{d}t} = -\mathrm{i}k_0(u_2+v_2) - \mathrm{i}\frac{k_0}{2}\Big[\eta_3u_1^\star + u_3\eta_1^\star + \eta_3v_1^\star + v_3\eta_1^\star +$$

$$+ \eta_5u_4^\star + u_5\eta_4^\star + \eta_5v_4^\star + v_5\eta_4^\star\Big] \quad (12)$$

$$\frac{\mathrm{d}\eta_3}{\mathrm{d}t} = -\mathrm{i}k_0(u_3+2v_3) - \mathrm{i}\frac{k_0}{2}\Big[\eta_2u_1 + u_2\eta_1 + 2\eta_2v_1 + 2v_2\eta_1\Big] \quad (13)$$

$$\frac{\mathrm{d}\eta_4}{\mathrm{d}t} = -\mathrm{i}k_0(2u_4-v_4) - \mathrm{i}\frac{k_0}{2}\Big[2\eta_5u_2^\star + 2u_5\eta_2^\star - \eta_5v_2^\star - v_5\eta_2^\star\Big] \quad (14)$$

$$\frac{\mathrm{d}\eta_5}{\mathrm{d}t} = -3\mathrm{i}k_0u_5 - \mathrm{i}\frac{k_0}{2}\Big[3\eta_4u_2 + 3u_4\eta_2\Big], \quad (15)$$

S1.2: Dynamics of amplitude and phases of $\mathcal{T}_5$ RSW system

The Fourier coefficients of the fields are complex variables so that we can define the amplitude and phases as $u_k = A_k\mathrm{e}^{\mathrm{i}\alpha_k}$, $v_k = B_k\mathrm{e}^{\mathrm{i}\beta_k}$ and $\eta_k = \Gamma_k\mathrm{e}^{\mathrm{i}\gamma_k}$. Using this representation, RSW equations (10)–(12) can be rewritten, thus obtaining a set of equations for both amplitudes and phases of the fields, which reads:

$$\frac{\mathrm{d}A_k}{\mathrm{d}t} - \frac{1}{2}\sum_{p,q}^{\Delta}\Big\{k_xA_pA_q\sin\Phi^{\alpha\alpha\alpha}_{-kpq} + q_yB_pA_q\sin\Phi^{\alpha\beta\alpha}_{-kpq} + p_yA_pB_q\sin\Phi^{\alpha\alpha\beta}_{-kpq}\Big\} =$$

$$= k_x\Gamma_k\sin(\alpha_k-\gamma_k) + \frac{B_k}{\mathrm{Ro}}\cos(\alpha_k-\beta_k) - \frac{k^2}{\mathrm{Re}}A_k +$$

$$+ \delta_{k,k_f}F_0\frac{\sqrt{2}}{2}\cos\left(\frac{\pi}{4}-\alpha_k\right) \quad (16)$$

$$A_k\frac{\mathrm{d}\alpha_k}{\mathrm{d}t} + \frac{1}{2}\sum_{p,q}^{\Delta}\Big\{k_yA_pA_q\cos\Phi^{\alpha\alpha\alpha}_{-kpq} + q_yB_pA_q\cos\Phi^{\alpha\beta\alpha}_{-kpq} + p_yA_pB_q\cos\Phi^{\alpha\alpha\beta}_{-kpq}\Big\} =$$

$$= k_x\Gamma_k\cos(\alpha_k-\gamma_k) - \frac{B_k}{\mathrm{Ro}}\sin(\alpha_k-\beta_k) + \delta_{k,k_f}F_0\frac{\sqrt{2}}{2}\sin\left(\frac{\pi}{4}-\alpha_k\right) \quad (17)$$

$$
\begin{aligned}
\frac{\mathrm{d}B_k}{\mathrm{d}t} &- \frac{1}{2}\sum_{p,q}^{\Delta}\left\{k_x B_p B_q \sin\Phi^{\beta\beta\beta}_{-kpq} + q_x A_p B_q \sin\Phi^{\beta\alpha\beta}_{-kpq} + p_x A_q B_p \sin\Phi^{\beta\beta\alpha}_{-kpq}\right\} = \\
&= k_y \Gamma_k \sin(\beta_k - \gamma_k) - \frac{A_k}{\mathrm{Ro}}\cos(\alpha_k - \beta_k) - \frac{k^2}{\mathrm{Re}}B_k \qquad (18)\\
B_k\frac{\mathrm{d}\beta_k}{\mathrm{d}t} &+ \frac{1}{2}\sum_{p,q}^{\Delta}\left\{k_x B_p B_q \cos\Phi^{\beta\beta\beta}_{-kpq} + q_x A_p B_q \cos\Phi^{\beta\alpha\beta}_{-kpq} p_x A_q B_p \cos\Phi^{\beta\beta\alpha}_{-kpq}\right\} = \\
&= k_y \Gamma_k \cos(\beta_k - \gamma_k) - \frac{A_k}{\mathrm{Ro}}\sin(\alpha_k - \beta_k) \qquad (19)
\end{aligned}
$$

$$
\begin{aligned}
\frac{\mathrm{d}\Gamma_k}{\mathrm{d}t} &- \frac{1}{2}\sum_{p,q}^{\Delta}\left\{k_x\left[A_q\Gamma_p \sin\Phi^{\gamma\gamma\alpha}_{-kpq} + A_p\Gamma_q\sin\Phi^{\gamma\alpha\gamma}_{-kpq}\right] + k_y\left[B_q\Gamma_p\sin\Phi^{\gamma\gamma\beta}_{-kpq} + B_p\Gamma_q\sin\Phi^{\gamma\beta\gamma}_{-kpq}\right]\right\} = \\
&= k_x\sin(\alpha_k - \gamma_k) + k_y\sin(\beta_k - \gamma_k) \qquad (20)\\
\Gamma_k\frac{\mathrm{d}\gamma_k}{\mathrm{d}t} &+ \frac{1}{2}\sum_{p,q}^{\Delta}\left\{k_x\left[A_q\Gamma_p \cos\Phi^{\gamma\gamma\alpha}_{-kpq} + A_p\Gamma_q\cos\Phi^{\gamma\alpha\gamma}_{-kpq}\right] + k_y\left[B_q\Gamma_p\cos\Phi^{\gamma\gamma\beta}_{-kpq} + B_p\Gamma_q\cos\Phi^{\gamma\beta\gamma}_{-kpq}\right]\right\} = \\
&= -k_x\cos(\alpha_k - \gamma_k) - k_y\cos(\beta_k - \gamma_k) \qquad (21)
\end{aligned}
$$

We defined the symbol $\Phi^{\alpha\beta\gamma}_{\pm kpq}$ which represents the three-phase couplings term, and is defined as:

$$
\Phi^{\alpha\beta\gamma}_{\pm kpq} = \pm\alpha_k + \beta_p + \gamma_q. \qquad (22)
$$

For completeness, we report here the same five-mode truncation using the Ansatz $u_k = A_k e^{i\alpha_k}$, $v_k = B_k e^{i\beta_k}$, $\eta_k = \Gamma_k e^{i\gamma_k}$ and $F_u = F_0(\sqrt{2}/2)(1+\mathrm{i})$

$$
\begin{aligned}
\frac{\mathrm{d}A_1}{\mathrm{d}t} &= \frac{k_0}{2}\left[-A_2B_3\sin\Phi^{\alpha\alpha\beta}_{-1-23} + 2B_2A_3\sin\Phi^{\alpha\beta\alpha}_{-1-23}\right] + \frac{B_1}{\mathrm{Ro}}\cos(\beta_1 - \alpha_1) - \frac{k_0^2}{\mathrm{Re}}A_1\\
\frac{\mathrm{d}A_2}{\mathrm{d}t} &= k_0\Gamma_2\sin(\gamma_2 - \alpha_2) + \frac{k_0}{2}\left[A_1A_3\sin\Phi^{\alpha\alpha\alpha}_{-1-23} - A_1B_3\sin\Phi^{\alpha\alpha\beta}_{-1-23} + 2B_1A_3\sin\Phi^{\beta\alpha\alpha}_{-1-23} + \right.\\
&+ \left. A_4A_5\sin\Phi^{\alpha\alpha\alpha}_{-2-45} + A_4B_5\sin\Phi^{\alpha\alpha\beta}_{-2-45}\right] + \frac{B_2}{\mathrm{Ro}}\cos(\beta_2 - \alpha_2) - 2\frac{k_0^2}{\mathrm{Re}}A_2\\
\frac{\mathrm{d}A_3}{\mathrm{d}t} &= k_0\Gamma_3\sin(\gamma_3 - \alpha_3) + \frac{k_0}{2}\left[A_1A_2\sin\Phi^{\alpha\alpha\alpha}_{12-3} + A_1B_2\sin\Phi^{\alpha\beta\alpha}_{12-3} + B_1A_2\sin\Phi^{\beta\alpha\alpha}_{12-3}\right] +\\
&+ \frac{B_3}{\mathrm{Ro}}\cos(\beta_3 - \alpha_3) - 5\frac{k_0^2}{\mathrm{Re}}A_3\\
\frac{\mathrm{d}A_4}{\mathrm{d}t} &= 2k_0\Gamma_4\sin(\gamma_4 - \alpha_4) + \frac{k_0}{2}\left[A_2A_5\sin\Phi^{\alpha\alpha\alpha}_{-2-45} - A_2B_5\sin\Phi^{\alpha\alpha\beta}_{-2-45}\right] + \frac{B_4}{\mathrm{Ro}}\cos(\beta_4 - \alpha_4) -\\
&- 5\frac{k_0^2}{\mathrm{Re}}A_4 + F_0\frac{\sqrt{2}}{2}\cos\left(\frac{\pi}{4} - \alpha_k\right)\\
\frac{\mathrm{d}A_5}{\mathrm{d}t} &= 3k_0\Gamma_5\sin(\gamma_5 - \alpha_5) + \frac{k_0}{2}\left[3A_2A_4\sin\Phi^{\alpha\alpha\alpha}_{24-5} + A_2B_4\sin\Phi^{\alpha\beta\alpha}_{24-5} - B_2A_4\sin\Phi^{\beta\alpha\alpha}_{24-5}\right] +\\
&+ \frac{B_5}{\mathrm{Ro}}\cos(\beta_5 - \alpha_5) - 9\frac{k_0^2}{\mathrm{Re}}A_5
\end{aligned}
$$

$$
\begin{aligned}
A_1\frac{\mathrm{d}\alpha_1}{\mathrm{d}t} &= -\frac{k_0}{2}\Big[-A_2B_3\cos\Phi^{\alpha\alpha\beta}_{-1-23}+2B_2A_3\cos\Phi^{\alpha\beta\alpha}_{12-3}\Big]+\frac{B_1}{\mathrm{Ro}}\sin(\beta_1-\alpha_1)\\
A_2\frac{\mathrm{d}\alpha_2}{\mathrm{d}t} &= -k_0\Gamma_2\cos(\gamma_2-\alpha_2)-\frac{k_0}{2}\Big[A_1A_3\cos\Phi^{\alpha\alpha\alpha}_{-1-23}-A_1B_3\cos\Phi^{\alpha\alpha\beta}_{-1-23}+\\
&+ 2B_1A_3\cos\Phi^{\beta\alpha\alpha}_{-1-23}+A_4A_5\cos\Phi^{\alpha\alpha\alpha}_{-2-45}+A_4B_5\cos\Phi^{\alpha\alpha\beta}_{-2-45}\Big]+\\
&+ \frac{B_2}{\mathrm{Ro}}\sin(\beta_2-\alpha_2)\\
A_3\frac{\mathrm{d}\alpha_3}{\mathrm{d}t} &= -k_0\Gamma_3\cos(\gamma_3-\alpha_3)-\frac{k_0}{2}\Big[A_1A_2\cos\Phi^{\alpha\alpha\alpha}_{12-3}+A_1B_2\cos\Phi^{\alpha\beta\alpha}_{12-3}+B_1A_2\cos\Phi^{\beta\alpha\alpha}_{12-3}\Big]+\\
&+ \frac{B_3}{\mathrm{Ro}}\sin(\beta_3-\alpha_3)\\
A_4\frac{\mathrm{d}\alpha_4}{\mathrm{d}t} &= -2k_0\Gamma_4\cos(\gamma_4-\alpha_4)-\frac{k_0}{2}\Big[A_2A_5\cos\Phi^{\alpha\alpha\alpha}_{-2-45}-A_2B_5\cos\Phi^{\alpha\alpha\beta}_{-2-45}\Big]+\frac{B_4}{\mathrm{Ro}}\sin(\beta_4-\alpha_4)+\\
&+ F_0\frac{\sqrt{2}}{2}\sin\left(\frac{\pi}{4}-\alpha_k\right)\\
A_5\frac{\mathrm{d}\alpha_5}{\mathrm{d}t} &= -3k_0\Gamma_5\cos(\gamma_5-\alpha_5)-\frac{k_0}{2}\Big[3A_2A_4\cos\Phi^{\alpha\alpha\alpha}_{24-5}+A_2B_4\cos\Phi^{\alpha\beta\alpha}_{24-5}-B_2A_4\cos\Phi^{\beta\alpha\alpha}_{24-5}\Big]+\\
&+ \frac{B_5}{\mathrm{Ro}}\sin(\beta_5-\alpha_5)
\end{aligned}
$$

$$
\begin{aligned}
\frac{\mathrm{d}B_1}{\mathrm{d}t} &= k_0\Gamma_1\sin(\gamma_1-\beta_1)+\frac{k_0}{2}\Big[B_2B_3\sin\Phi^{\beta\beta\beta}_{-1-23}-B_2A_3\sin\Phi^{\beta\beta\alpha}_{-1-23}+A_2B_3\sin\Phi^{\beta\alpha\beta}_{-1-23}\Big]-\\
&- \frac{A_1}{\mathrm{Ro}}\cos(\alpha_1-\beta_1)-\frac{k_0^2}{\mathrm{Re}}B_1\\
\frac{\mathrm{d}B_2}{\mathrm{d}t} &= k_0\Gamma_2\sin(\gamma_2-\beta_2)+\frac{k_0}{2}\Big[B_1B_3\sin\Phi^{\beta\beta\beta}_{-1-23}+A_1B_3\sin\Phi^{\alpha\beta\beta}_{-1-23}+B_4B_5\sin\Phi^{\beta\beta\beta}_{-2-45}+\\
&+ 3A_4B_5\sin\Phi^{\beta\alpha\beta}_{-2-45}-2B_4A_5\sin\Phi^{\beta\beta\alpha}_{-2-45}\Big]-\frac{A_2}{\mathrm{Ro}}\cos(\alpha_2-\beta_2)-2\frac{k_0^2}{\mathrm{Re}}B_2\\
\frac{\mathrm{d}B_3}{\mathrm{d}t} &= 2k_0\Gamma_3\sin(\gamma_3-\beta_3)+\frac{k_0}{2}\Big[B_1B_2\sin\Phi^{\beta\beta\beta}_{12-3}+A_1B_2\sin\Phi^{\alpha\beta\beta}_{12-3}\Big]-\frac{A_3}{\mathrm{Ro}}\cos(\alpha_3-\beta_3)-5\frac{k_0^2}{\mathrm{Re}}B_3\\
\frac{\mathrm{d}B_4}{\mathrm{d}t} &= -k_0\Gamma_4\sin(\gamma_4-\beta_4)+\frac{k_0}{2}\Big[-B_2B_5\sin\Phi^{\beta\beta\beta}_{-2-45}-B_2A_5\sin\Phi^{\beta\beta\alpha}_{-2-45}+3A_2B_5\sin\Phi^{\alpha\beta\beta}_{-2-45}\Big]-\\
&- \frac{A_4}{\mathrm{Ro}}\cos(\alpha_4-\beta_4)-5\frac{k_0^2}{\mathrm{Re}}B_4\\
\frac{\mathrm{d}B_5}{\mathrm{d}t} &= \frac{k_0}{2}\Big[B_2A_4\sin\Phi^{\beta\alpha\beta}_{24-5}+2A_2B_4\sin\Phi^{\alpha\beta\beta}_{24-5}\Big]-\frac{A_5}{\mathrm{Ro}}\cos(\alpha_5-\beta_5)-9\frac{k_0^2}{\mathrm{Re}}B_5
\end{aligned}
$$

$$
\begin{aligned}
B_1\frac{\mathrm{d}\beta_1}{\mathrm{d}t} &= -k_0\Gamma_1\cos(\gamma_1-\beta_1)-\frac{k_0}{2}\Big[B_2B_3\cos\Phi^{\beta\beta\beta}_{-1-23}-B_2A_3\cos\Phi^{\beta\beta\alpha}_{-1-23}+A_2B_3\cos\Phi^{\beta\alpha\beta}_{-1-23}\Big]-\\
&- \frac{A_1}{\mathrm{Ro}}\sin(\alpha_1-\beta_1)\\
B_2\frac{\mathrm{d}\beta_2}{\mathrm{d}t} &= -k_0\Gamma_2\cos(\gamma_2-\beta_2)-\frac{k_0}{2}\Big[B_1B_3\cos\Phi^{\beta\beta\beta}_{-1-23}+A_1B_3\cos\Phi^{\alpha\beta\beta}_{-1-23}+B_4B_5\cos\Phi^{\beta\beta\beta}_{-2-45}+\\
&+ 3A_4B_5\cos\Phi^{\beta\alpha\beta}_{-2-45}-2B_4A_5\cos\Phi^{\beta\beta\alpha}_{-2-45}\Big]-\frac{A_2}{\mathrm{Ro}}\sin(\alpha_2-\beta_2)\\
B_3\frac{\mathrm{d}\beta_3}{\mathrm{d}t} &= -2k_0\Gamma_3\cos(\gamma_3-\beta_3)-\frac{k_0}{2}\Big[B_1B_2\cos\Phi^{\beta\beta\beta}_{12-3}+A_1B_2\cos\Phi^{\alpha\beta\beta}_{12-3}\Big]-\frac{A_3}{\mathrm{Ro}}\sin(\alpha_3-\beta_3)\\
B_4\frac{\mathrm{d}\beta_4}{\mathrm{d}t} &= k_0\Gamma_4\sin(\gamma_4-\beta_4)+\frac{k_0}{2}\Big[-B_2B_5\cos\Phi^{\beta\beta\beta}_{-2-45}-B_2A_5\cos\Phi^{\beta\beta\alpha}_{-2-45}+3A_2B_5\cos\Phi^{\alpha\beta\beta}_{-2-45}\Big]-\\
&- \frac{A_4}{\mathrm{Ro}}\sin(\alpha_4-\beta_4)\\
B_5\frac{\mathrm{d}\beta_5}{\mathrm{d}t} &= -\frac{k_0}{2}\Big[B_2A_4\cos\Phi^{\beta\alpha\beta}_{24-5}+2A_2B_4\cos\Phi^{\alpha\beta\beta}_{24-5}\Big]-\frac{A_5}{\mathrm{Ro}}\sin(\alpha_5-\beta_5)
\end{aligned}
$$

$$
\begin{aligned}
\frac{\mathrm{d}\Gamma_1}{\mathrm{d}t} &= k_0B_1\sin(\beta_1-\gamma_1)+\frac{k_0}{2}\Big[B_2\Gamma_3\sin\Phi^{\gamma\beta\gamma}_{-1-23}+\Gamma_2B_3\sin\Phi^{\gamma\gamma\beta}_{-1-23}\Big]\\
\frac{\mathrm{d}\Gamma_2}{\mathrm{d}t} &= k_0A_2\sin(\alpha_2-\gamma_2)+k_0B_2\sin(\beta_2-\gamma_2)+\\
&+ \frac{k_0}{2}\Big[\Gamma_3A_1\sin\Phi^{\alpha\gamma\gamma}_{-1-23}+\Gamma_1A_3\sin\Phi^{\gamma\gamma\alpha}_{-1-23}+\Gamma_3B_1\sin\Phi^{\beta\gamma\gamma}_{-1-23}+\\
&+ \Gamma_1B_3\sin\Phi^{\gamma\gamma\beta}_{-1-23}+\Gamma_5A_4\sin\Phi^{\gamma\alpha\gamma}_{-2-45}+A_5\Gamma_4\sin\Phi^{\gamma\gamma\alpha}_{-2-45}+\\
&+ \Gamma_5B_4\sin\Phi^{\gamma\beta\gamma}_{-2-45}+\Gamma_4B_5\sin\Phi^{\gamma\gamma\beta}_{-2-45}\Big]\\
\frac{\mathrm{d}\Gamma_3}{\mathrm{d}t} &= k_0A_3\sin(\alpha_3-\gamma_3)+k_0B_3\sin(\beta_3-\gamma_3)+\frac{k_0}{2}\Big[\Gamma_2A_1\sin\Phi^{\alpha\gamma\gamma}_{12-3}+\Gamma_1A_2\sin\Phi^{\gamma\alpha\gamma}_{12-3}+\\
&+ 2\Gamma_2B_1\sin\Phi^{\beta\gamma\gamma}_{12-3}+\Gamma_1B_2\sin\Phi^{\gamma\beta\gamma}_{12-3}\Big]\\
\frac{\mathrm{d}\Gamma_4}{\mathrm{d}t} &= 2k_0A_4\sin(\alpha_4-\gamma_4)-k_0B_4\sin(\beta_4-\gamma_4)+\frac{k_0}{2}\Big[2A_2\Gamma_5\sin\Phi^{\alpha\gamma\gamma}_{-2-45}+2\Gamma_2A_5\sin\Phi^{\gamma\gamma\alpha}_{-2-45}-\\
&- B_2\Gamma_5\sin\Phi^{\beta\gamma\gamma}_{-2-45}-\Gamma_2B_5\sin\Phi^{\gamma\gamma\beta}_{-2-45}\Big]\\
\frac{\mathrm{d}\Gamma_5}{\mathrm{d}t} &= 9k_0A_5\sin(\alpha_5-\gamma_5)+\frac{k_0}{2}\Big[3A_2\Gamma_4\sin\Phi^{\alpha\gamma\gamma}_{24-5}+3\Gamma_2A_4\sin\Phi^{\gamma\alpha\gamma}_{24-5}\Big]
\end{aligned}
$$

$$
\begin{aligned}
\Gamma_1 \frac{\mathrm{d}\gamma_1}{\mathrm{d}t} &= -k_0 B_1 \cos(\beta_1 - \gamma_1) - \frac{k_0}{2}\Big[B_2 \Gamma_3 \cos\Phi^{\gamma\beta\gamma}_{-1-23} + \Gamma_2 B_3 \cos\Phi^{\gamma\gamma\beta}_{-1-23}\Big] \\
\Gamma_2 \frac{\mathrm{d}\gamma_2}{\mathrm{d}t} &= -k_0 A_2 \cos(\alpha_2 - \gamma_2) - k_0 B_2 \cos(\beta_2 - \gamma_2) + \\
&+ \frac{k_0}{2}\Big[\Gamma_3 A_1 \cos\Phi^{\alpha\gamma\gamma}_{-1-23} + \Gamma_1 A_3 \cos\Phi^{\gamma\gamma\alpha}_{-1-23} + \Gamma_3 B_1 \cos\Phi^{\beta\gamma\gamma}_{-1-23} + \\
&+ \Gamma_1 B_3 \cos\Phi^{\gamma\gamma\beta}_{-1-23} + \Gamma_5 A_4 \cos\Phi^{\gamma\alpha\gamma}_{-2-45} + A_5 \Gamma_4 \cos\Phi^{\gamma\gamma\alpha}_{-2-45} + \\
&+ \Gamma_5 B_4 \cos\Phi^{\gamma\beta\gamma}_{-2-45} + \Gamma_4 B_5 \cos\Phi^{\gamma\gamma\beta}_{-2-45}\Big] \\
\Gamma_3 \frac{\mathrm{d}\gamma_3}{\mathrm{d}t} &= -k_0 A_3 \cos(\alpha_3 - \gamma_3) + k_0 B_3 \cos(\beta_3 - \gamma_3) - \\
&- \frac{k_0}{2}\Big[\Gamma_2 A_1 \cos\Phi^{\alpha\gamma\gamma}_{12-3} + \Gamma_1 A_2 \cos\Phi^{\gamma\alpha\gamma}_{12-3} + \\
&+ 2\Gamma_2 B_1 \cos\Phi^{\beta\gamma\gamma}_{12-3} + \Gamma_1 B_2 \cos\Phi^{\gamma\beta\gamma}_{12-3}\Big] \\
\Gamma_4 \frac{\mathrm{d}\gamma_4}{\mathrm{d}t} &= -2k_0 A_4 \cos(\alpha_4 - \gamma_4) + k_0 B_4 \cos(\beta_4 - \gamma_4) - \\
&- \frac{k_0}{2}\Big[2A_2 \Gamma_5 \cos\Phi^{\alpha\gamma\gamma}_{-2-45} + 2\Gamma_2 A_5 \cos\Phi^{\gamma\gamma\alpha}_{-2-45} - \\
&- B_2 \Gamma_5 \cos\Phi^{\beta\gamma\gamma}_{-2-45} - \Gamma_2 B_5 \cos\Phi^{\gamma\gamma\beta}_{-2-45}\Big] \\
\Gamma_5 \frac{\mathrm{d}\gamma_5}{\mathrm{d}t} &= -9k_0 A_5 \cos(\alpha_5 - \gamma_5) - \frac{k_0}{2}\Big[3A_2 \Gamma_4 \cos\Phi^{\alpha\gamma\gamma}_{24-5} + 3\Gamma_2 A_4 \cos\Phi^{\gamma\alpha\gamma}_{24-5}\Big]
\end{aligned}
$$
