# Supplementary material for: Route to chaos and resonant triads interaction in a truncated rotating nonlinear shallow–water model
Source: PLoS One. 2024 Aug 9;19(8):e0305534. doi: 10.1371/journal.pone.0305534 (PMC11315320; doi:10.1371/journal.pone.0305534)
Supplement: Attachment — Submitted filename: Reply3.pdf [file pone.0305534.s002.pdf]

Answers to referee III

We would like to acknowledge the Referees for their valuable comments and suggestions, which contributed to the improvement of the new version of the manuscript. Below are the points raised by the Referee I and the relative comment/answer. All modifications to the manuscript have been highlighted in bold.

Q1: The abstract of the manuscript needs to be supplemented with appropriate key quantitative results to enhance its persuasiveness. For example, it was mentioned that "It was observed that the duration of phase stability diminishes with an increase in injected energy, culminating in the onset of chaos within the phase components at high energy levels.".

A1: As suggested by the referee, we added a few words in the abstract in order to make clear the focus of the work related to the Galerkin models:
[...] *have been rigorously examined through the construction of an autonomous five-mode Galerkin truncated system employing complex variables,* ***useful in investigating how large/meso-scales are destabilized and how their dynamics evolves and transits to chaos****. This investigation revealed* [...]

Q2: The sentences in lines 4-6 that "Moreover, their interplay is related to essential processes in atmospheric and oceanic sciences, such as transport and exchange of moisture, heat, gaseous tracers or salinity, and momentum, depending on the which of boundary layer being considered." should be supported by citing some works.

A2: Done, three useful citations were added on lines 4-6:
[...] *depending on the which of boundary layer being considered [1–3] (*i.e. *oceanic or atmospheric)* [...].

Q3: However, how the model was implemented and through what tools? These details also need to be appropriately displayed.

A3: As explained in the manuscript, starting from line 104, all equations have been derived analytically from the authors starting from the RSW complete system (equations 1 and 2). Moreover, as written on line 101, all numerical runs are performed using a classical fourth-order Runge-Kutta time marching scheme, with a fixed nondimensional timestep $\Delta t = 0.125$. The equations were solved numerically with a Fortran code. As noted by the referee, since the chosen solution method is present in all textbooks, no further details have been added to the text. In order to help the reader, we have added a book reference explaining the method. the new reference is:
Butcher J. C., The Numerical Analysis of Ordinary Differential Equations: Runge-Kutta and General Linear Methods. Wiley, 1987.

Q4: How applicable is the model constructed in the second part to the relevant research content in this manuscript? The reviewer believes that it is necessary for the model to be validated before application.

A4: The reduced phase model was derived analytically from equations 1 and 2 as the full RSW model. In particular, this new model allows us to analyze in greater detail the temporal phase dynamics of the various modes composing the system and search for eventual phase locking periods or other features. We agree with the referee when he suggests that further studies are needed for other possible applications so we have added a few words in the conclusions in order to explicitly state this concept:
[···] *Even if further efforts and studies are required to fully understand the detailed nature of RSW turbulence on geostrophic scales, this work opens novel approaches to study the dynamics and the transition to chaos via multiple bifurcations and intermittent transitions, in RSW systems, within the general framework of dynamical systems theory.*

Q5: In the manuscript, it is not friendly to the review process if the author places the main text, figures and tables separately. It is recommended to insert the figures and tables into the corresponding positions of the text when submitting the revised manuscript.

A5: The figures have been added at the end of the manuscript as specified in the manuscript

preparation guidelines. In fact, the guide specified that figures should not be added manuscript file, as reported below:
Do not include figures in the main manuscript file. Each figure must be prepared and submitted as an individual file.

Q6: Why is there a significant difference in the variation trend of the curve with T when the C value is high compared to the curve when the C value is low in Figure 13?

A6: The difference is mainly due to how the system distributes the energy injected by the forcing during the various phase-locking periods. In particular, for low $\mathcal{C}$ values, the duration of the phase jumps is longer, so the drift induced in the phase dynamic is weaker since it accumulates fewer pass jumps. On the other hand, for the case where $\mathcal{C}$ is large, the system redistributes energy more efficiently, and since the phase locking periods have a significantly shorter duration, a significantly higher frequency of phase jumps is observed. Their successive accumulation gives different and much higher values in the dynamics of $\Phi(T\,\mathrm{Ro}^{-1})$.

[1] Qingchao Li, Jin Liu, Shiming Wang, Ying Guo, Xiaoying Han, Qiang Li, Yuanfang Cheng, Zhuo Dong, Xianzhong Li, and Xiaodong Zhang. Numerical insights into factors affecting collapse behavior of horizontal wellbore in clayey silt hydrate-bearing sediments and the accompanying control strategy. *Ocean Engineering*, 297:117029, 2024. ISSN 0029-8018. doi: https://doi.org/10.1016/j.oceaneng.2024.117029. URL `https://www.sciencedirect.com/science/article/pii/S0029801824003664`.

[2] Roland B. Stull. *An introduction to boundary layer meteorology*. Atmospheric and Oceanographic Sciences Library. Kluwer Academic Publishers, Dordrecht, 1 edition, 1988. ISBN 978-94-009-3027-8.

[3] B. J. H. Van de Wiel, A. F. Moene, O. K. Hartogensis, H. A. R. De Bruin, and A. A. M. Holtslag. Intermittent turbulence in the stable boundary layer over land. part iii: A classification for observations during cases-99. *Journal of the Atmospheric Sciences*, 60(20):2509–2522, 2003. doi: 10.1175/1520-0469(2003)060⟨2509:ITITSB⟩2.0.CO;2. URL https://doi.org/10.1175/1520-0469(2003)060<2509:ITITSB>2.0.CO;2.
